# Rainbow Vectors for Broad-Range Bacterial Fluorescence Labeling

**DOI:** 10.1371/journal.pone.0146827

**Published:** 2016-03-03

**Authors:** Mariette Barbier, F. Heath Damron

**Affiliations:** West Virginia University School of Medicine, Department of Microbiology, Immunology and Cell Biology, Morgantown, West Virginia, United States of America; East Carolina University School of Medicine, UNITED STATES

## Abstract

Since their discovery, fluorescent proteins have been widely used to study protein function, localization or interaction, promoter activity and regulation, drug discovery or for non-invasive imaging. They have been extensively modified to improve brightness, stability, and oligomerization state. However, only a few studies have focused on understanding the dynamics of fluorescent proteins expression in bacteria. In this work, we developed a set plasmids encoding 12 fluorescent proteins for bacterial labeling to facilitate the study of pathogen-host interactions. These broad-spectrum plasmids can be used with a wide variety of Gram-negative microorganisms including *Escherichia coli*, *Pseudomonas aeruginosa*, *Burkholderia cepacia*, *Bordetella bronchiseptica*, *Shigella flexneri* or *Klebsiella pneumoniae*. For comparison, fluorescent protein expression and physical characteristics in *Escherichia coli* were analyzed using fluorescence microscopy, flow cytometry and *in vivo* imaging. Fluorescent proteins derived from the *Aequorea Victoria* family showed high photobleaching, while proteins form the *Discosoma sp*. and the *Fungia coccina* family were more photostable for microscopy applications. Only E2-Crimson, mCherry and mKeima were successfully detected for *in vivo* applications. Overall, E2-Crimson was the fastest maturing protein tested in *E*. *coli* with the best overall performance in the study parameters. This study provides a unified comparison and comprehensive characterization of fluorescent protein photostability, maturation and toxicity, and offers general recommendations on the optimal fluorescent proteins for *in vitro* and *in vivo* applications.

## Introduction

Pathogenic and non-pathogenic bacteria colonize complex ecosystems. The study of their lifestyle and interaction with these environments often requires the use of non-invasive tools. The green fluorescent protein (GFP), isolated more than 50 years ago from the jellyfish *Aequorea victoria* [1], can be used to observe real-time biological processes in these environments. This protein has been extensively used to study protein function and localization, promoter activity and regulation, drug discovery, and non-invasive imaging. In microbiology, fluorescent proteins have often been used to label and study microorganisms in complex systems, including *in vivo* models of infection such as plants [2], fish [3,4], and mammals [5]. Over the years, numerous GFP variants have been generated to increase levels of fluorescence emission, change the degree of oligomerization, or switch the excitation and emission spectra. The protein GFP*mut3* for example was derived from GFP to increase its compatibility with standard filters used commonly for green fluorescing dyes such as fluorescein isothiocyanate (FITC) and facilitate its use for flow cytometry [6]. Since the discovery of GFP, other fluorescent proteins have been isolated from numerous organisms such as *Discosoma* sp., *Montipora* sp., *Anthozoa*, *Trachyphyllia geoffroyi* or *Fungia coccina* [7–9]. These proteins have also been extensively modified to improve brightness, stability, oligomerization and expression levels in various hosts. However, only a few studies have focused on understanding the dynamics of fluorescent proteins expression in bacteria.

Due to the advantages of fluorescent proteins, numerous vectors have been created to use fluorescent proteins as reporters, fusion proteins, or cell labeling [4,10–13]. However, there is still a lack of broad-range molecular tools for the labeling of Gram-negative bacteria with fluorescent protein covering the entire light spectrum. To provide better tools for microbiologists interested in the use of fluorescent proteins, we developed a set of broad-spectrum plasmids encoding 12 different fluorescent proteins to facilitate the study of bacteria in complex environments, including pathogen-host interactions. Proteins covering a wide range of excitation and emission wavelengths from blue to far red (excitation: 399-610nm; emission: 476-649nm) were cloned in the vectors pUCP20T and pUCP30T. We also performed an extensive characterization of the different characteristics and applications of these proteins to offer general recommendations on optimal fluorescent proteins use for *in vitro* and *in vivo* applications.

## Materials and Methods

### Bacterial strains and plasmids

In this study, we used *E*. *coli* strain JM109 53323 (American Type Culture Collection, Manassas, VA) for inducible fluorescent protein expression (*lacI*^*q*^*)* and E. cloni 10G (Lucigen, Middleton, WI) for constitutive expression (Δ*lac*X74). Additionally, the plasmids generated in this study were transferred and expressed in *Pseudomonas aeruginosa* (Type strain PAO1 obtained from Dr. M. Vasil, University of Colorado, USA), *Salmonella enterica* ATCC 14028, *Burkholderia cepacia* ATCC 25416, *Bordetella bronchiseptica* ATCC 4617, *Klebsiella pneumoniae* ATCC 13883 and *Shigella flexneri* ATCC 12022. Plasmids pUCP20T, pUCP30T, pUC18C-mini-Tn7T-Gm-*ecfp*, pUC18C-mini-Tn7T-Gm-gfpmut3, pUC18C-mini-Tn7T-Gm-eyfp and pUC18C-mini-Tn7T-Gm-dsRedExpress were kindly provided by Dr. H. P. Schweizer. Other plasmids listed in S1 Table were obtained from Addgene (Cambridge, MA). Plasmids were introduced in the strains listed above by electroporation. Electrocompetent cells were prepared using a method previously described [14]. Bacteria were plated on Lysogeny agar (LA) supplemented with 100 μg/ml carbenicillin for *E*. *coli*, 10 μg/ml gentamycin for *E*. *coli*, *S*. *enterica*, *K*. *pneumoniae* and *S*. *flexneri*, or 100 μg/ml gentamycin for *P*. *aeruginosa and B*. *cepacia*.

### Construction of the rainbow vectors

Fluorescent proteins were amplified by polymerase chain reaction (PCR) using the templates listed in S1 Table. Kappa high-fidelity DNA Taq polymerase (Kappa Biosystems, Wilmington, MA) and the primers listed in S2 Table were used for amplification. These primers were designed with an EcoRI restriction site on the 5’ of the amplification product and a BamHI restriction site on the 3’ end of the product to facilitate direct digestion of the PCR products after amplification. PCR fragment were purified using a MiniElute reaction cleanup kit (Qiagen, Germantown, MD) and digested using EcoRI and BamHI (New England Biolabs, Ipswich, MA). Fragments were then purified again using MiniElute reaction cleanup kit. The plasmids pUCP20T and pUCP30T [15] were purified by miniprep, linearized with EcoRI and BamHI, and purified using MiniElute reaction cleanup kit. Digested fluorescent protein fragments were then ligated into linearized pUCP20T using Fast-Link DNA ligase (Epicenter, Charlotte, NC) to generate the pUCP20T and pUCP30T rainbow series (S2 Table). Sequence insertion was confirmed by Sanger sequencing using M13 primers. The vectors generated in this study are listed in Table 1. All vector sequences have been deposited on NCBI and accession numbers are detailed in Table 1.

**Table 1:** Plasmids used and generated in this study.

Plasmid name	Accession n°	Antibiotic resistance	References
pUCP20T	U33750	Ampicillin	[16]
pUCP30T	U33752	Gentamycin	[15]
pUCP20T-*ecfp*	KT878729	Ampicillin	This study
pCUP20T-*tsapphire*	KT878737	Ampicillin	This study
pUCP20T-*gfpmut3*	KT878731	Ampicillin	This study
pUCP20T-*eyfp*	KT878730	Ampicillin	This study
pCUP20T-*mko1*	KU215429	Ampicillin	This study
pUCP20T-*morange*	KT878734	Ampicillin	This study
pUCP20T-*tdTomato*	KT878736	Ampicillin	This study
pUCP20T-*dsRedExpress*	KT878743	Ampicillin	This study
pUCP20T-*mCherry*	KT878732	Ampicillin	This study
pUCP20T-*mKeima*	KT878733	Ampicillin	This study
pUCP20T-*E2Crimson*	KT878728	Ampicillin	This study
pUCP20T-*mPlum*	KT878735	Ampicillin	This study
pUCP30T-*gfpmut3*	KT878740	Gentamycin	This study
pUCP30T-*eyfp*	KT878739	Gentamycin	This study
pUCP30T-*mKo1*	KT878742	Gentamycin	This study
pUCP30T-*E2Crimson*	KT878738	Gentamycin	This study
pUCP30T-*mKeima*	KT878741	Gentamycin	This study

### Sequence alignment

Nucleotide sequences from the amplified proteins were obtained from the NCBI and Addgene databases. Sequences were translated to protein *in silico* and aligned using CLC Main Workbench v7.6.1 (Qiagen, Germatown, MD). A radial tree was generated with this software using Neighbor Joining and Jukes-Cantor nucleotide distance measurement and a bootstrap analysis with 100 replicates.

### Effects of fluorescent protein expression on bacterial growth fitness

*E*. *coli* strains E. cloni 10G harboring the pUCP20T rainbow plasmids described in Table 1 were grown overnight in 5 ml Lysogeny broth (LB) with 100 μg/ml carbenicillin. Fresh Lysogeny broth with 100 μg/ml carbenicillin was inoculated with a dilution factor of 1:100 and incubated at 37°C under constant shaking in a flat bottom clear 96 well plate. Absorbance was measured at regular intervals using a Spectramax I3 fluorescence microplate reader (Molecular Devices, Sunnyvale, CA). Optical densities were corrected for 1 cm path length. Experiments were performed in triplicate and data were statistically analyzed using unpaired two-tailed Student *t-*tests and the software Prizm 6.0 (GraphPad, La Jolla, CA).

### Induction of fluorescent protein expression

*E*. *coli* JM109 harboring the pUCP20T rainbow plasmids described in Table 1 were grown overnight in 5 ml LB with 100 μg/ml carbenicillin. Twenty five ml of fresh LB with 100 μg/ml carbenicillin in 125 ml flasks were inoculated with a dilution factor of 1:100 and incubated at 37°C under constant shaking. After 12 H of incubation, 25 μl of a solution of 40 μM Isopropyl β-D-1-thiogalactopyranoside (IPTG) or phosphate buffered saline (PBS) as control were added to each flask. Induction was maintained until the end of the experiment (13 H). Flasks were incubated at 37°C under constant shaking and samples were removed every 1 to 2 hours to measure absorbance and fluorescence using the Spectramax I3 fluorescence microplate reader. Different excitation and emission wavelengths were selected for eCFP (Ex: 433/15 nm, Em: 476/25 nm), T-Sapphire (Ex: 499/9 nm, Em: 511/15 nm), GFP*mut3* (Ex: 510/9 nm, Em: 530/15 nm), eYFP (Ex: 510/9 nm, Em: 530/15 nm), mKO1 (Ex: 548/9 nm, Em: 568/15 nm), mOrange (Ex: 548/9 nm, Em: 568/15 nm), tdTomato (Ex: 554/9 nm, Em: 585/15 nm), dsRedExpress (Ex: 554/9 nm, Em: 585/15 nm), mCherry (Ex: 587/9 nm, Em: 610/15 nm), mKeima (Ex: 440/9 nm, Em: 620/15 nm) and E2-Crimson (Ex: 611/9 nm, Em: 646/15 nm). Experiments were performed in triplicate. Data were normalized using the absorbance at 600 nm and statistically analyzed using one sample *t-*tests and the software Prizm 6.0.

### Photostability

*E*. *coli* JM109 harboring the rainbow plasmids described in Table 1 were grown overnight on LA with 100 μg/ml carbenicillin and 40 μg/ml IPTG. Strains were smeared on a glass microscope slide, covered with a coverslip and imaged at x100 with immersion oil using a Zeiss Axioscop fluorescence microscope (Carl Zeiss, Oberkochen, Germany). Excitation of the slides was performed using a Carl Zeiss Arc Lamp set on 100 W, 3.18 A and 31.4 V. Slides with eCFP, T-Sapphire, *GFPmut3* and eYFP were imaged with a filter XF100-2 (Excitation: 475nm, Emission: 535nm) while slides with mKO1, mOrange, tdTomato, dsRedExpress, mCherry, mKeima, E2-Crimson and mPlum were imaged with a filter XF414 (Excitation: 560nm, Emission: 645nm) (Omega Optical, Brattleboro, Vermont). Slides were excited continuously and images were acquired with an AxioCam HRc (Carl Zeiss, Oberkochen, Germany) and the software AxioVision LE (Carl Zeiss, Oberkochen, Germany) at regular intervals. Image maximal, minimal, and average intensity were measured in 9 independent areas of 200x200px using ImageJ (rsbweb.nih.gov/ij/). Photobleaching half-life was calculated as the time necessary for each fluorescent protein to lose 50% of its initial brightness. Experiments were performed in triplicate and statistically analyzed using unpaired two-tailed Student t-tests and the software Prizm 6.0.

### Flow cytometry analysis

*E*. *coli* strain JM109 harboring pUCP20T empty vector or rainbow vectors described in Table 1 was grown in LB supplemented with 100 μg/ml of carbenicillin and 40 μM IPTG for 16H. Bacteria were washed three times with PBS and finally resuspended in PBS. Bacterial suspensions were analyzed using an LSRFortessa (BD) flow cytometer and Diva 8.0 software. For each sample, 100,000 events were collected. The flow cytometer was equipped with four lasers: 405 nm OBIS LX laser, 488 nm Sapphire laser, 561 nm Sapphire laser and 628 nm OEM laser. The emission bandpass filters in front of the 405 nm laser detectors were 450/50 nm and 525/50 nm. The emission bandpass filters in front of the 488 nm laser detectors were 515/30 nm, 610/20 nm and 695/40 nm. The emission bandpass filters in front of the 561 nm laser detectors were 585/15 nm, 610/20 nm, 660/20 nm, 695/40 nm and 780/60 nm. The emission bandpass filters in front of the 628 nm laser detectors 670/30 nm, 730/45 nm and 780/60 nm. Voltages on the detectors was set so that the MFI of bacteria without vector was between 70 and 100 for all fluorescent parameters. These setting were used to determine the mean fluorescence intensity (MFI) and the best excitation and emission wavelengths for each fluorescent protein. Spectral overlap was then determined at these wavelengths for each protein. Data are presented as the average of three technical replicates and were analyzed with an unpaired two-tailed *t*-test and the software Prizm 6.0.

### Fluorescence imaging using an *in vivo* imaging system

*E*. *coli* strain E. cloni 10G harboring the plasmids listed in Table 1 were growth for 18H on LA supplemented with 100 μg/ml of carbenicillin. Plates were imaged using an *in vivo* imaging systems (IVIS spectrum, Perkin Elmer, Waltham, MA) at excitation and emission wavelengths ranging from 430 nm to 760 nm. For phantom mouse imaging, these strains were grown in LB supplemented with 100 μg/ml of carbenicillin and serial dilutions were performed. Ten μl of bacterial suspension were loaded into a capillary which was inserted into the phantom mouse (Perkin Elmer). The mouse was imaged at various excitation and emission wavelengths ranging from 430 nm to 760 nm for each fluorescent protein tested using the IVIS spectrum. The measurement of photons in a specific region of interest were performed in triplicate and background was subtracted from these values. The number of CFUs was determined by plating bacterial suspensions on LA supplemented with 100 μg/ml of carbenicillin at the appropriate dilutions. Data were analyzed with an unpaired two-tailed *t*-test and the software Prizm 6.0.

## Results

### Construction of rainbow vectors

**Fig 1:**
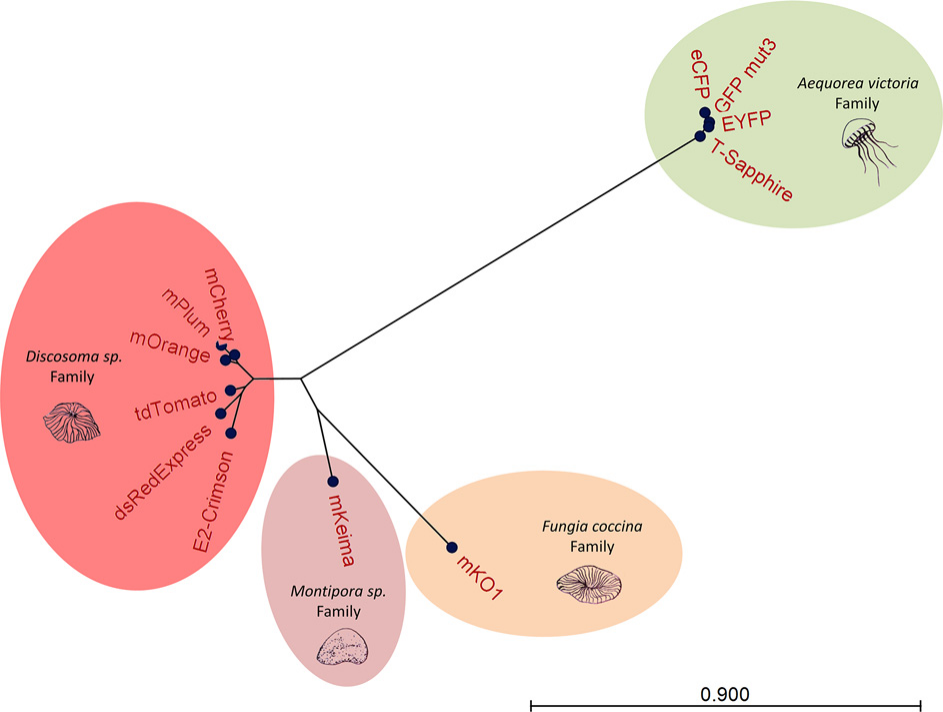
Radial Neighbor Joining tree of the fluorescent protein sequences selected in this study. Fluorescent protein sequence alignments were analyzed using a Neighbor Joining radial tree. Analysis was performed using CLC Main Workbench v7.6.1. The distance between the nodes is indicated below the graph.

Fluorescent proteins have been extensively used in the last five decades for the study of biological processes. These proteins have been modified for use *in vitro* and more than a hundred different proteins are now available. As a result, it is often difficult to determine which fluorescent protein is best adapted for a specific application. In this study, we generated a set of broad-range multi-copy vectors for efficient fluorescent protein expression and bright fluorescence labeling in Gram-negative bacteria. To this end, we selected 12 different fluorescent proteins isolated from various organisms (Fig 1). In each group, various proteins with different degrees of brightness, spectrum and stability optimization were chosen for comparison. Two sets of vectors based on pUCP20T and pUCP30T [15] were generated by inserting the sequence of 12 fluorescent proteins in the multiple cloning site of these vectors (Table 1). The rainbow vectors have the features of the pUCP vector family [15,17,18], including the pBR322 origin, ori1600, ampicillin or gentamycin antibiotic resistance markers, and the *rep* gene encoding the replication-controlling protein. These vectors also encode an oriT region for conjugation-mediated plasmid transfer. Fluorescent protein expression is controlled by the *lacZ* promoter (P_*lacZ*_) for induced expression in *E*. *coli* [19]. Additionally, we were able to show that these vectors can be used for constitutive expression of fluorescent proteins in other microorganisms such as *Pseudomonas aeruginosa*, *Burkholderia cepacia*, *Klebsiella pneumoniae*, *Salmonella enterica*, *Shigella flexneri* or *Bordetella bronchiseptica* (data not shown).

### Effect of fluorescence protein expression on bacterial fitness

**Fig 2:**
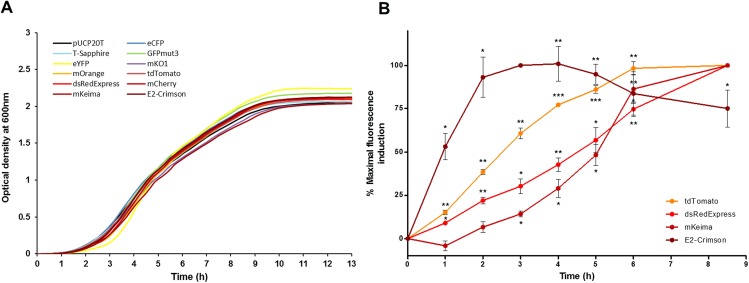
Fluorescent protein expression in *E*. *coli*: effects on bacterial fitness and protein maturation. (A) *E*. *coli* strain E. cloni 10G harboring the plasmids listed in Table 1 were grown for 13 H in LB under constant shaking in a flat bottom clear 96 well plate. Absorbance at 600 nm was measured every 15 minutes using a Spectramax I3 fluorescence microplate reader (Molecular Devices, Sunnyvale, CA). (B) *E*. *coli* strain JM109 harboring the rainbow plasmids listed in Table 1 were grown in culture flasks under constant shaking for 12 H in LB. Fluorescent protein expression was then induced by adding 40 μM IPTG or PBS as control. Induction was maintained until the end of the experiment. Changes in absorbance and fluorescence were monitored using a Spectramax I3. Data are represented as percentage of the maximal fluorescence value reached during induction. Data were normalized using the absorbance at 600nm and analyzed with one sample *t*-test and the software Prizm 6.0. Significant changes in fluorescence compared to the non-induced control are denoted with an asterisk (*: *p*<0.05; **: *p*<0.01; ***: *p*<0.001).

Expression of high protein levels can often alter bacterial growth, decrease fitness and generate toxicity. The effect of fluorescent protein expression in *E*. *coli* was tested on bacterial growth. *E*. *coli* strain E. cloni 10G harboring the pUCP20T rainbow vectors for constitutive fluorescent protein expression was grown in LB in presence of 100 μg/ml of carbenicillin at 37°C in a 96 well plate under constant shaking, and absorbance was measured at regular intervals. No differences were observed between the control strain harboring the empty pUCP20T control vector and the strains expressing the fluorescent proteins on the optical density (Fig 2A) or colony forming units (data not shown) during growth in LB at 37°C. These results suggest that expression of fluorescent proteins using these vectors does not negatively affect the growth fitness and viability of *E*. *coli in vitro*.

### Expression, maturation and stability

Fluorescent proteins have been optimized to facilitate expression at 37°C in eukaryotic cells. In prokaryotes, differences in transcription and translation machineries, enzymes, and chaperones can substantially influence protein expression, folding, and maturation efficiency. To understand the dynamics of fluorescent protein synthesis and folding *in vitro*, we compared the maturation speed of four closely related red fluorescent proteins. *E*. *coli* strain JM109 harboring the rainbow vectors encoding for tdTomato, dsRedExpress, mKeima and E2-Crimson were grown in LB until reaching stationary phase. Fluorescent protein expression was then induced with IPTG and changes in fluorescence were detected using a fluorescence microplate reader. Fluorescence values were corrected for changes in absorbance at 600nm. IPTG induction did not alter significantly bacterial growth (data not shown). Induction of tdTomato, dsRedExpress and E2-Crimson expression was significantly detected within the first hour of induction (Fig 2B). mKeima levels were not significantly detected until 3 H of induction. In most cases, maximal fluorescence was not achieved until 8.5 H. E2-Crimson was the fastest maturating protein, reaching maximal fluorescence levels in 3 H (Fig 2B).

### Photostability

**Fig 3:**
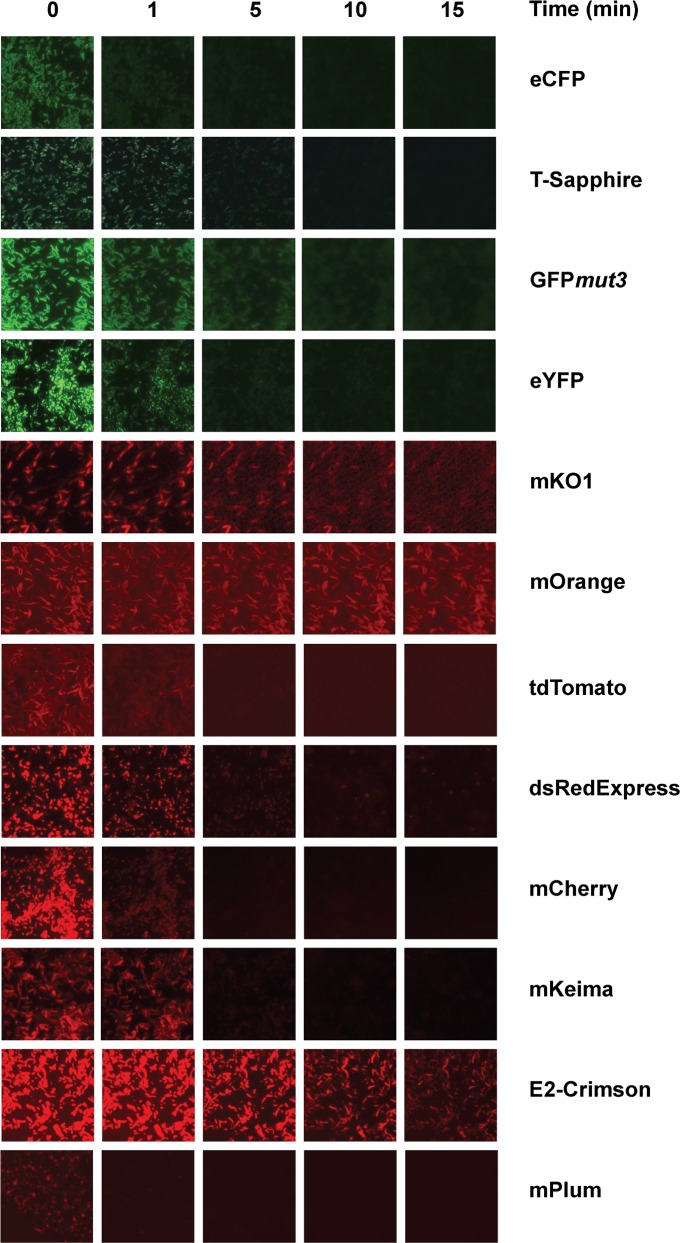
Photobleaching during fluorescence microscopy imaging. *E*. *coli* JM109 harboring the pUCP20T derived plasmids described in Table 1 were smeared on a microscopy slide, covered with a coverslip and imaged at x100 with immersion oil using a Zeiss Axioscop fluorescence microscope. Slides were excited continuously and images were acquired at regular intervals. Experiments were performed in triplicate and representative images are presented here.

**Fig 4:**
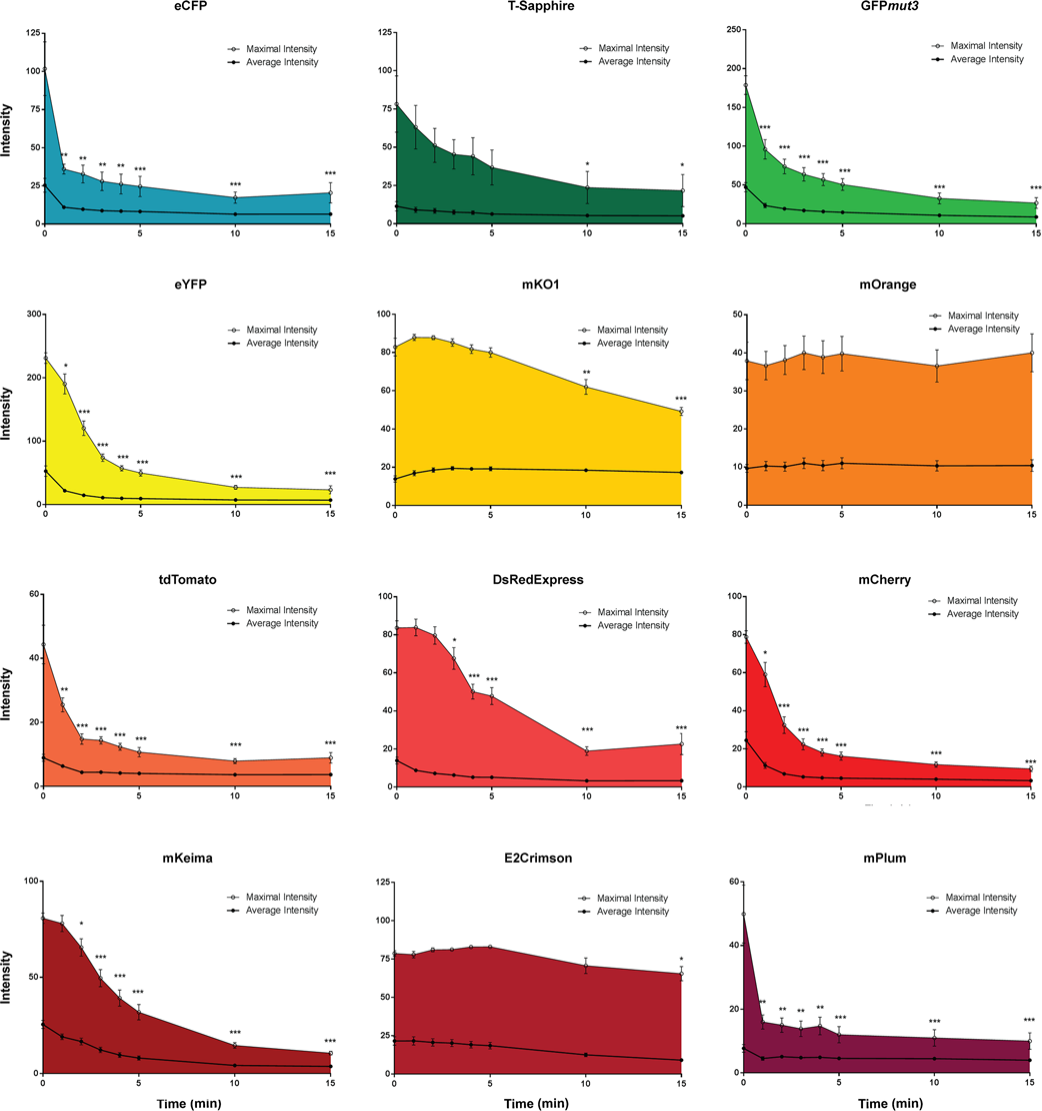
Sensitivity to photobleaching. E. coli JM109 harboring the pUCP20T derived plasmids described in Table 1 were smeared on a microscopy slide, covered with a coverslip and imaged at x100 with immersion oil using a Zeiss Axioscop fluorescence microscope. Slides were excited continuously and images were acquired at regular intervals. Fluorescence intensity was measured using ImageJ. Experiments were performed in triplicate and data statistically analyzed using unpaired two-tailed Student *t*-tests and the software Prizm 6.0.

Fluorescent proteins are sensitive to light and photobleaching upon excitation. The rate of photobleaching varies greatly between different fluorescent proteins and is crucial for efficient microscopy imaging. Photobleaching has been extensively characterized using purified fluorescent proteins, however, environmental factors can affect the properties of fluorescent proteins and little is known on the photobleaching dynamics of these proteins when expressed in bacterial cells. Here, we measured the photobleaching curves of 12 fluorescent proteins expressed in *E*. *coli* strain JM109 induced with IPTG under conditions simulating standard fluorescent microscopy imaging. Bacterial smears were photobleached under continuous UV illumination with specific excitation and emission filters and imaged periodically to generate bleaching curves. Half-life photobleaching was measured as the time necessary for each fluorescent protein to lose 50% of its initial brightness (Table 2). Results indicate that the proteins studied can be separated in two groups according to their resistance to photobleaching. eCFP, T-sapphire, GFP*mut3*, eYFP, tdTomato, dsRedExpress, mCherry, mKeima and mPlum had a high sensitivity to photobleaching with short half-life and a fast initial photobleaching (Figs 3 and 4). The proteins with the highest resistance to photobleaching were mKO1, mOrange and E2-Crimson (Figs 3 and 4). All the proteins from the *A*. *victoria* family were highly sensitive to photobleaching. However, there was a lot of disparity in the *Discosoma sp*. family. Overall, fluorescent proteins in the orange to red spectrum were more stable than those in the blue to yellow spectrum. Interestingly, during this analysis, we also observed that *E*. *coli* cells expressing dsRedExpress, mCherry and mPlum appeared shorter than those from the control harboring pUCP20T (Fig 3).

**Table 2:** Physical and chemical characteristics of the fluorescent proteins used in this study.

Fluorescent protein	Ex. (nm)	Em. (nm)	Extinction coefficient (M^-1^cm^-1^)	Quantum yield	Brightness*	pH sensitivity (pKa)	Structure	Range	Photobleaching half-life in *E*. *coli* (s)	Origin	Ref.
eCFP	433	476	26,000	0.4	10.4	4.7	Tetramer	Cyan	52	*Aequorea victoria*	[20]
T-Sapphire	399	511	44,000	0.6	26.4	4.9	Monomer/Weak dimer	UV excited green	244	*Aequorea victoria*	[21]
GFP*mut3*	505	511					Tetramer	Green	69	*Aequorea victoria*	[6]
eYFP	514	527	84,000	0.61	51.24	6.5	Tetramer	Yellow	134	*Aequorea victoria*	[20]
mKO1	548	561	73,700	0.45	33.2	<5.0	Dimer	Orange	> 900	*Fungia coccina*	[22]
mOrange	548	563	71,000	0.69	48.99	6.5	Monomer	Orange	> 900	*Discosoma sp*.	[7]
tdTomato	554	581	138,000	0.69	95.22	4.7	Tandem dimer	Red	118	*Discosoma sp*.	[7]
dsRedExpress	554	586	33,800	0.44	14.872		Tetramer	Red	357	*Discosoma sp*.	[23]
mCherry	587	610	72,000	0.22	15.84	<4.5	Monomer	Red	107	*Discosoma sp*.	[7]
mKeima	440	620	14,400	0.24	3.5	6.5	Monomer	Far red	219	*Montipora sp*.	[24]
E2-Crimson	611	646	126,000	0.23	28.98	4.5		Far red	> 900	*Discosoma sp*.	[25]
mPlum	590	649	29,300	0.1	2.93	5.5	Monomer	Far red	65	*Discosoma sp*.	[26]

*Brightness: Molar Extinction Coefficient ×Fluorescence Quantum Yield / 1000.

### Flow cytometry applications

**Fig 5:**
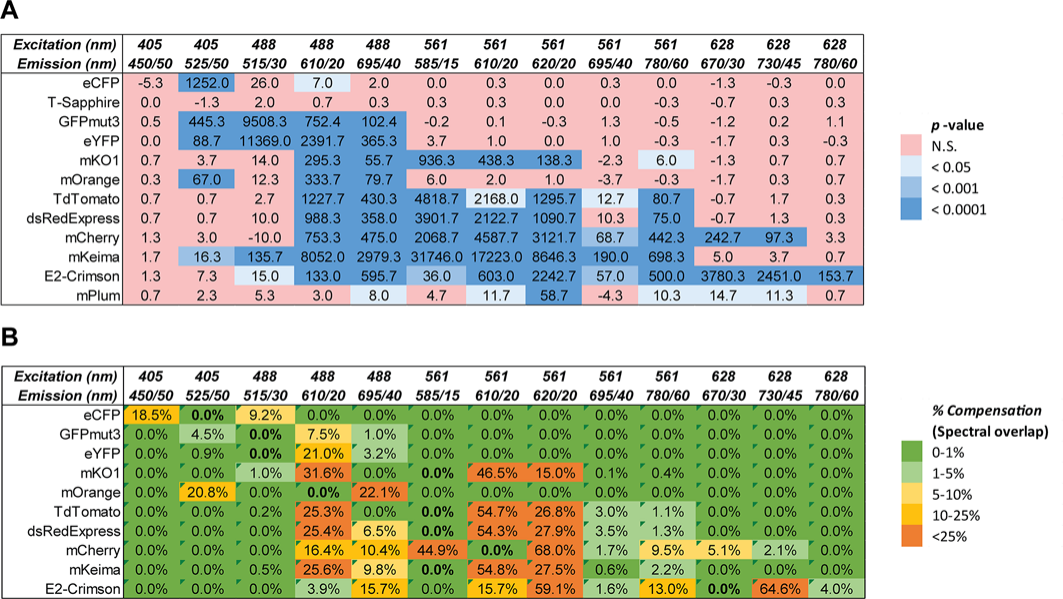
Flow cytometry analysis of *E*. *coli* labeled with fluorescent proteins. Bacterial suspensions of *E*. *coli* JM109 harboring the plasmids described in Table 1 were analyzed by flow cytometry using an LSRFortessa (BD). A. Bacterial fluorescence was measured with a constant voltage at the excitation wavelengths of 405 nm, 488 nm, 561 nm and 628 nm and the emission wavelengths of 450/50 nm, 515/30 nm, 525/50 nm, 585/15 nm, 610/20 nm, 620/20 nm, 670/30 nm, 695/40 nm, 730/45 nm and 780/60 nm. Data were analyzed with an unpaired two-tailed *t*-test and the software Prizm 6.0. Fluorescence values significantly higher than those of the unlabeled control bacteria are labeled in blue. No significant changes are shown in red. B. Spectral overlap represented as percentage of compensation was measured for each fluorescent protein at the excitation and emission wavelengths indicated in bold.

Flow cytometry can be used for cell detection using fluorescence labeling. This method can also be used for the detection of free bacteria as well as bacteria bound to eukaryotic cells. However, not all fluorescent proteins provide channel-specific labeling for multi-color applications. Here, we tested *E*. *coli* strain E. cloni 10G labeled with twelve fluorescent proteins using flow cytometry. The fluorescence of each protein was measured with thirteen different combinations of excitation and emission wavelengths. The best excitation/emission wavelength settings were determined for each protein (Fig 5B). T-Sapphire was not detected by flow cytometry, while mPlum and mOrange fluorescence levels were low compared to other fluorescent proteins tested. Proteins from the *A*. *victoria* family emitting in the blue to yellow range did not require significant compensation to be detected in combination with other fluorescent proteins. However, proteins emitting in the red range such as mKO1, TdTomato, dsRedExpress, mCherry, mKeima and E2-Crimson were significantly detected with various combination of lasers and emission filters (Fig 5A) and required greater levels of compensation to correct for the spectral overlap of these proteins (Fig 5B). These results indicate that the signal of many fluorescent proteins can be detected in multiple channels, making the analysis and channel compensation more difficult. Overall, most of these proteins can be used in combination in a single assay as long as the appropriate compensation settings are used.

### Use of fluorescent protein for *in vivo* imaging

**Fig 6:**
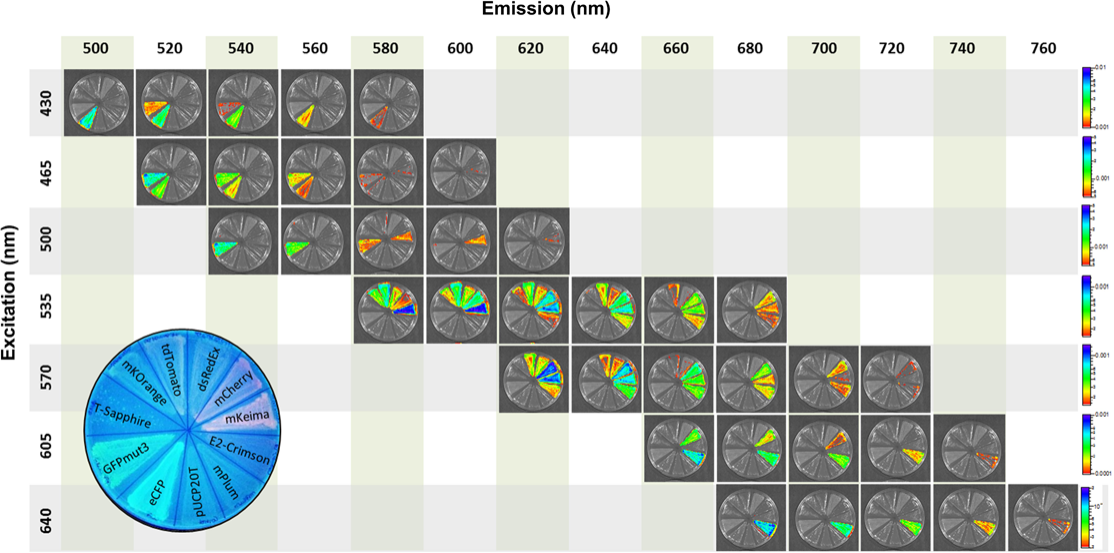
Bacterial fluorescence detection using an *in vivo* imaging system (IVIS). *E*. *coli* strain JM109 harboring the plasmids listed in Table 1 were growth for 18 H on LA with 40 μM IPTG and 100 μg/ml carbenicillin. Plates were imaged using an IVIS Spectrum at the excitation and emission wavelength indicated on the figure. Fluorescence intensity is indicated on the right. The location of the strains harboring each plasmid on the plate is indicated on the bottom left.

For the study of pathogen-host interactions, fluorescent proteins can facilitate the detection of bacterial pathogens during infection using IVIS. However, the high absorbance of hemoglobin and skin melanin often impedes their detection. Here, we first studied the range of detection of various fluorescent proteins in an *in vitro* setting using a Perkin Elmer IVIS Spectrum. *E*. *coli* strain E. cloni 10G was grown on LA and imaged at various excitation and emission wavelengths ranging from 420 nm to 760 nm. Using this equipment, all proteins but T-Sapphire and mPlum were detected. eCFP and GFP*mut3* were easily differentiated from each other (Fig 6). However, mKO1, tdTomato, dsRedExpress, mCherry and mKeima were detected at similar excitation and emission wavelengths, indicating that their use in combination is unadvisable using IVIS (Fig 6). E2-Crimson, mKeima and mCherry showed the highest fluorescence intensities in the red range (Fig 6).

**Fig 7:**
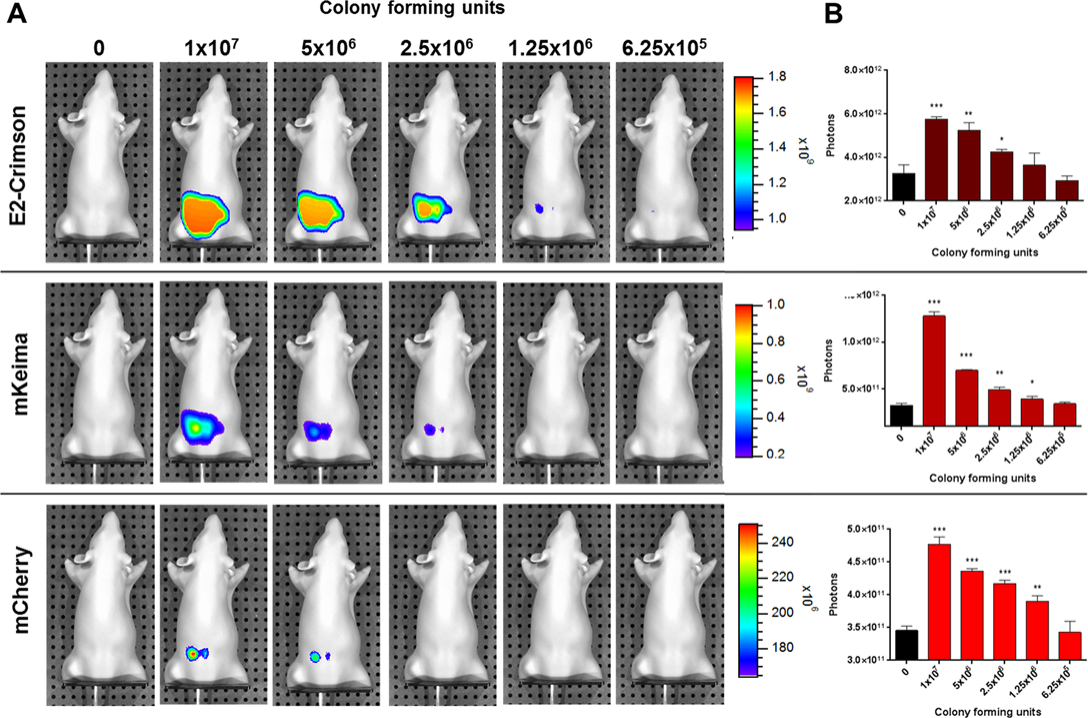
Determination of fluorescence detection limits *in vivo* using a phantom mouse. 6.25x10^5^ to 1x10^7^ CFUs of *E*. *coli* strain E. cloni 10G were loaded into a capillary tube and inserted into the phantom mouse. The mouse was imaged at excitation and emission wavelengths ranging from 430 nm to 760 nm for each fluorescent protein tested using the IVIS spectrum (A). The scale for the number of photons detected is indicated on the right. (B). Photons detected in the regions of interest for each amount of bacteria. Measurement were performed in triplicate and data were analyzed using an unpaired two-tailed t-test and the software Prizm 6.0. (*: *p*<0.05; **: *p*<0.01; ***: *p*<0.001).

Various known numbers of CFUs were then inserted in a phantom mouse to detect bacterial fluorescence in an *in vivo*-like setting. This mouse-shaped phantom is made of polyurethane material to simulate the optical properties of live tissue, and measure the effects of tissue fluorescence absorption [27]. Only E2-Crimson, mKeima and mCherry were successfully detected in the phantom mouse (Fig 7). The detection of E2-Crimson and mKeima was comparable. Bacterial concentrations under 2.5x10^6^ CFU were not detectable (Fig 7). These results indicate that the use of blue to orange fluorescence proteins for deep tissue *in vivo* applications is highly unadvisable. Furthermore, this type of application is only recommended in infection models where the expected number of fluorescent bacteria is higher than 2.5x10^6^ CFU in the tissue of interest.

## Discussion

The successful use of fluorescent proteins in a specific experimental setting depends on various factors. The protein should be expressed at levels sufficient for detection in the system of interest but without being toxic, be bright for detection above autofluorescence levels, and stable enough to allow measurements during the duration of the experiment [28]. Additionally, when various fluorescent proteins are used in the same experiment, minimal crosstalk should be recorded on their excitation and emission channels. These characteristics are specific to the models of expression and experimental setting used. Here, we compared the expression, toxicity, photostability and crosstalk of various fluorescent proteins using diverse imaging methods in the bacterium *E*. *coli*. Fluorescent proteins expression can be toxic in eukaryotic and prokaryotic cells, especially when expressed at high levels or for an extended period of time [29]. In this study, we did not detect any changes in viability or growth fitness in fluorescent protein expressing bacteria compared to the control. However, changes in bacterial size were detected in bacteria expressing some of the fluorescent proteins from the *Discosoma* sp. family (dsRedExpress, mCherry and mPlum). Similar results had been previously obtained for dsRedExpress in *E*. *coli* [29]. E2-Crimson, another protein from the *Discosoma* sp. family engineered to emit fluorescence on the far red spectrum and to have reduced toxicity [23,25], did not alter the morphology of *E*. *coli*. These results suggest that while *E*. *coli* successfully expresses fluorescent proteins at levels sufficient for fluorescence imaging, some of these proteins might exert negative effects on cell physiology and metabolism. Further studies are required to determine the cellular effects of the expression of these fluorescent proteins on *E*. *coli*. Photostability studies performed here indicate that many fluorescent proteins tested bleach rapidly under continuous exposure to UV light. The experimental setup reproduced standard conditions with typical excitation for an epifluorescence microscope. While photobleaching half-life might be different using other imaging systems such as laser scanning confocal microscopy, our results indicate that mKO1, mOrange and E2-Crimson are the most optimal proteins tested providing images of high fluorescence and contrast over a long imaging period. Flow cytometry and plate imaging of the fluorescent proteins expressed in *E*. *coli* reinforce the importance of using the right filter for fluorescence imaging. A high level of crosstalk was observed on most of the channels tested and extensive optimization is required to detect specific signal in multiple labeling experiments.

The results obtained in this study confirm that the use of fluorescent proteins to label bacteria for *in vivo* fluorescence application can be difficult. While most fluorescent proteins were detected on an agar plate, indicating the potential use of these proteins for surface imaging, only fluorescent proteins emitting in the red to far-red range were successfully detected in the phantom mouse. Furthermore, these proteins were only detected when bacteria were present at loads higher than 2.5x10^6^ CFU. These caveats limit the use of fluorescent proteins in deep-tissue infection models with low bacterial load. To circumvent this problem, near-infrared fluorescent proteins have been developed to increase signal detection *in vivo* [30,31]. However, many of these near-infrared fluorescent proteins also require co-expression of a heme-oxygenase to produce biliverdin Ixα. For eukaryotic applications, this method generates a strong fluorescence signal but requires more genetic engineering for applications in prokaryotes.

Overall, this study emphasizes the importance of selecting the most appropriate fluorescent protein for each application. T-Sapphire and mPlum were only successfully detected by fluorescence microcopy, potentially due to the low brightness and high sensitivity to photobleaching of these proteins. E2-Crimson showed best overall performance with a fast maturation, high contrast and low photobleaching for microscopy applications, and a far red spectrum allowing for easy discrimination from other fluorescent proteins by flow cytometry and *in vivo* imaging.

## Supporting Information

S1 Table Plasmids used as template to amplify fluorescent proteins. (DOCX)

S2 Table Oligonucleotides used in this study. (DOCX)
