# Supplementary material for: Rainbow Vectors for Broad-Range Bacterial Fluorescence Labeling
Source: PLoS One. 2016 Mar 3;11(3):e0146827. doi: 10.1371/journal.pone.0146827 (PMC4777285; doi:10.1371/journal.pone.0146827)
Supplement: S1 Table — (DOCX) [file pone.0146827.s001.docx]

***S1 Table:*** Plasmids used as template to amplify fluorescent proteins

| **Fluorescent protein** | **Plasmids** | **Reference** |
| --- | --- | --- |
| eCFP | pUC18C-mini-Tn7T-Gm-*ecfp* | [10] |
| T-Sapphire | pFA6a-link-yETSapphire-CaUra3 | [32] |
| GFP*mut3* | pUC18C-mini-Tn7T-Gm-*gfpmut3* | [10] |
| eYFP | pUC18C-mini-Tn7T-Gm-*eyfp* | [10] |
| mKO1 | pQC mKorange IX | [33] |
| mOrange | pBAD-LSSmOrange | [34] |
| tdTomato | pQC TdTomato IX | [33] |
| dsRedExpress | pUC18C-mini-Tn7T-Gm-*dsRedExpress* | [10] |
| mCherry | pFPV-mCherry | [35] |
| mKeima | pFA6a-link-yomKeima-CaURA3 | [16] |
| E2-Crimson | YIPlac204TKC-E2-Crimson-HDEL | [25] |
| mPlum | Hsp70-mplum | [36] |

***Additional references:***

32. Sheff MA, Thorn KS. Optimized casettes for fluorescent protein tagging in *Saccharomyces cerevisiae*. Yeast. 2004;21(8):661-70.

33. Beier KT, Samson ME, Matsuda T, Cepko CL. Conditional expression of the TVA repectors allows clonal analysis of descendents from Cre-expressing progenitor cells. Developmental biology. 2011;353(2):309-20.

34. Shcherbakova DM, Hink MA, Joosen L, Gadella TW, Verkusha VV. An orange fluorescent protein with a large Strokes shift for single-excitation multicolor FCCS and FRET imaging. Journal of the American Chemical Society. 2012;134(18):7913-23.

35. Drecktrah D, Levine-Wilkinson S, Dam T, Winfree S, Knodler LA, Schroer TA, et al. Dynamic behavior of *Salmonella*-induced membrane tubules in epithelial cells. Traffic. 2008;9(12):2117-29.

36. de la Rosa X, Santalucia T, Fortin PY, Purroy J, Calvo M, Salas-Perdomo A, et al. In vivo imaging of induction of heat-shock protein-70 gene expression with fluorescence reflectance imaging and intravital confocal microscopy following brain ischaemia in reporter mice. Eur J Nucl Med Mol Imaging. 2013;40: 426–438. doi:10.1007/s00259-012-2277-7
