# Supplementary material for: Rainbow Vectors for Broad-Range Bacterial Fluorescence Labeling
Source: PLoS One. 2016 Mar 3;11(3):e0146827. doi: 10.1371/journal.pone.0146827 (PMC4777285; doi:10.1371/journal.pone.0146827)
Supplement: S2 Table — (DOCX) [file pone.0146827.s002.docx]

***S2 Table:*** Oligonucleotides used in this study

| **Fluorescent protein** | **5' primer** | **3' primer** |
| --- | --- | --- |
| eCFP | AGTCGAATTCGGCGCGCCAAGAAGGTCTAGAATTAAAGAGGAG | AGTCGGATCCAGCTTGGATTCTCACCAA |
| T-Sapphire | AGTCGAATTCGGCGCGCCAAGAAGGATGTCTAAAGGTGAAGAA | AGTCGGATCCTTATTTGTACAATTCATC |
| GFP*mut3* | AGTCGAATTCGGCGCGCCAAGAAGGTCTAGAATTAAAGAGGAG | AGTCGGATCCAGCTTGGATTCTCACCAA |
| eYFP | AGTCGAATTCGGCGCGCCAAGAAGGTCTAGAATTAAAGAGGAG | AGTCGGATCCAGCTTGGATTCTCACCAA |
| mKO1 | AGTCGAATTCGGCGCGCCAAGAAGGATGGTGAGTGTGATTAAA | AGTCGGATCCTCAGCAATGAGCTACTGC |
| mOrange | AGTCGAATTCGGCGCGCCAAGAAGGATGGGGAGCCACCATCA | AGTCGGATCCTTACTTGTACAGCTCGTC |
| tdTomato | AGTCGAATTCGGCGCGCCAAGAAGGATGGTGAGCAAGGGCGAG | AGTCGGATCCTTACTTGTACAGCTCGTC |
| dsRedExpress | AGTCGAATTCGGCGCGCCAAGAAGGTCTAGAATTAAAGAGGAG | AGTCGGATCCAGCTTGGATTCTCACCAA |
| mCherry | AGTCGAATTCGGCGCGCCAAGAAGGATGGTGAGCAAGGGCGAG | AGTCGGATCCTTACTTGTACAGCTCGTC |
| mKeima | AGTCGAATTCGGCGCGCCAAGAAGGATGGTTTCTGTGATCGCT | AGTCGGATCCTTACCCTAATAGAGAATG |
| E2-Crimson | AGTCGAATTCGGCGCGCCAAGAAGGATGTTTTTCAACAGACTA | AGTCGGATCCTTACAATTCGTCGTGCTT |
| mPlum | AGTCGAATTCGGCGCGCCAAGAAGGATGGTGAGCAAGGGCGAG | AGTCGGATCCTTAGGCGCCGGTGGAGTG |
